# Non-metastatic Nephrogenic Hepatic Dysfunction (Stauffer Syndrome) and Syndrome of Inappropriate Antidiuretic Hormone Secretion (SIADH) in a Patient With Renal Cell Carcinoma Coinciding With Liposarcoma

**DOI:** 10.7759/cureus.55714

**Published:** 2024-03-07

**Authors:** Sabahuddin L Hajjar, Hezborn M Magacha, Shahnawaz N Notta, David Joseph

**Affiliations:** 1 Internal Medicine, East Tennessee State University James H. Quillen College of Medicine, Johnson City, USA; 2 Internal Medicine, East Tennessee State University, Johnson City, USA; 3 Nephrology, Veterans Affairs Medical Center, Mountain Home, Johnson City, USA

**Keywords:** paraneoplastic, renal cell carcinoma, hepatic dysfunction, stauffer syndrome, siadh

## Abstract

Stauffer syndrome is a non-metastatic, nephrogenic, hepatic dysfunction syndrome that is linked to extrahepatic paraneoplastic tumors. It manifests with varying clinical signs that include jaundice, anicteric transaminitis, elevated alkaline phosphatase, thrombocytosis, elevated erythrocyte sedimentation rate, prolonged prothrombin time, and, in some cases, hepatosplenomegaly in the absence of hepatobiliary obstruction. Stauffer syndrome is mostly associated with renal cell carcinoma, but research shows other solid malignancies are implicated with this syndrome. Stauffer syndrome is characterized by elevated liver function tests, specifically those that indicate the presence of cholestasis with or without hepatosplenomegaly. The abnormality is not due to tumor infiltration but rather indirect paraneoplastic effects that are poorly understood. Additionally, emerging literature also supports the association of syndrome of inappropriate antidiuretic hormone secretion (SIADH) secondary to malignancy in the setting of elevated interleukin-6. In this article, we present the case of a 76-year-old patient with SIADH and abnormalities in liver function tests in the context of Stauffer syndrome tied to renal cell carcinoma coinciding with liposarcoma.

## Introduction

Stauffer syndrome is a rare syndrome that manifests in many types of cancers, including renal cell carcinoma (RCC), bladder cancer, metastatic prostate carcinoma, and malignant lymphoproliferative diseases [1]. Historically named non-metastatic, nephrogenic, hepatic dysfunction syndrome, it manifests with reversible anicteric transaminitis, elevated alkaline phosphatase, thrombocytosis, elevated erythrocyte sedimentation rate, prolonged prothrombin time, and, in some cases, hepatosplenomegaly in the absence of hepatobiliary obstruction. More than 100 cases of Stauffer syndrome have been reported since it was first discovered in 1961. Most cases are associated with RCC with an incidence of 3%-6%, followed by soft tissue sarcomas or prostate cancer; however, the incidence remains unclear [2]. Stauffer syndrome was first described with RCC associated with hepatosplenomegaly and abnormality of liver function with characteristic features of cholestatic disease pattern, with the key feature being the absence of metastasis to the liver. The mechanism of how Stauffer syndrome occurs is poorly understood as the incidence remains very low. However, the literature suggests it may be due to increased interleukin-6 (IL-6) from RCC [3]. The increased IL-6 appears to have an immuno-neuroendocrine effect on cells in the posterior pituitary causing the release of vasopressin by binding both soluble and insoluble IL-6R, which activate complex cascades of transcription factors and affect the production of transport proteins made by biliary cells of the liver [4]. A recent case study illustrated a patient diagnosed with Stauffer syndrome undergoing corticosteroid treatment for suspected giant arteritis. Subsequently, the patient exhibited reduced cholestasis and other inflammatory indicators. This observation implies the involvement of IL-6 and other inflammatory markers in the progression of this syndrome [5-7].

Here, we present the case of a 76-year-old male who presented with progressive fatigue, loss of appetite, confusion, and weight loss over five months. The evaluation revealed a renal mass, hyponatremia, and abnormal liver function tests (LFTs). Earlier, he had hyponatremia and transaminitis. The renal mass was removed, confirming clear cell renal carcinoma. Although symptoms improved temporarily, a left thigh mass was found. Hyponatremia persisted, and LFTs worsened, leading to a presentation for failure to thrive.

## Case presentation

**Figure 1:**
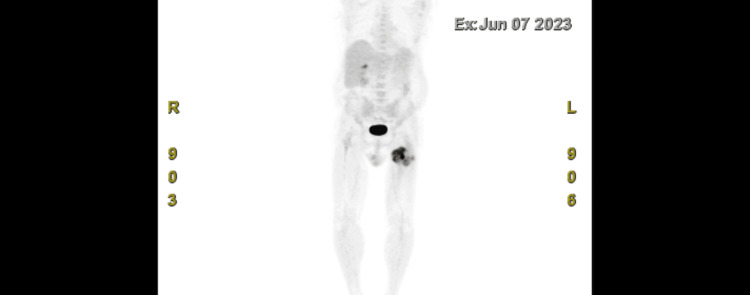
Positron emission tomography scan showing a 12 cm mass on the left thigh.

**Figure 2:**
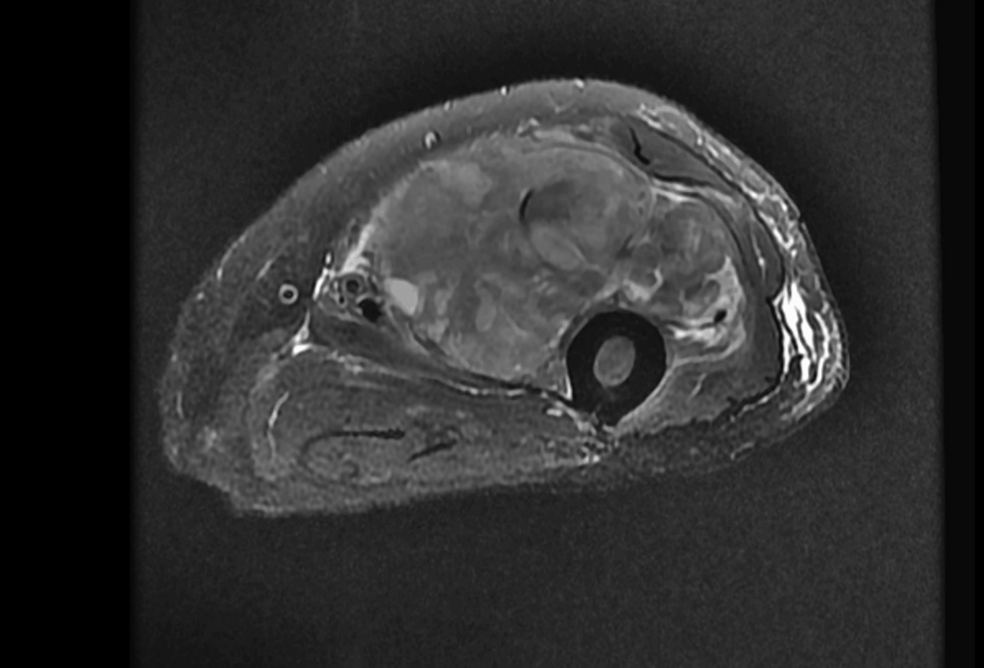
Magnetic resonance imaging showing a 12.2 cm mass deep in the sartorius muscle close to the rectus femoris.

A 76-year-old male with a history of prostate cancer, chronic obstructive pulmonary disease, atrial fibrillation treated with ablation, hypertension, a left nephrectomy due to RCC, and a left thigh mass presented to the hospital due to diminished oral intake and persistent diarrhea. On admission, he was found to have hyponatremia with a sodium level of 129 mEq/L and elevated LFTs with alkaline phosphatase (ALP) of 240 U/L and aspartate transaminase (AST) of 52 U/L. Previous workup of the hyponatremia revealed elevated urine osmolality that did not respond to intravenous (IV) fluid administration, which was consistent with syndrome of inappropriate antidiuretic hormone secretion (SIADH). A positron emission tomography scan previously done by oncology showed a mass on his left thigh with no other metastasis (Figure 1), and magnetic resonance imaging (MRI) showed a 12.2 cm mass deep in the sartorius muscle close to the rectus femoris muscle and calcifications near the neurovascular bundle of the profunda femoris artery (Figure 2).

**Figure 3:**
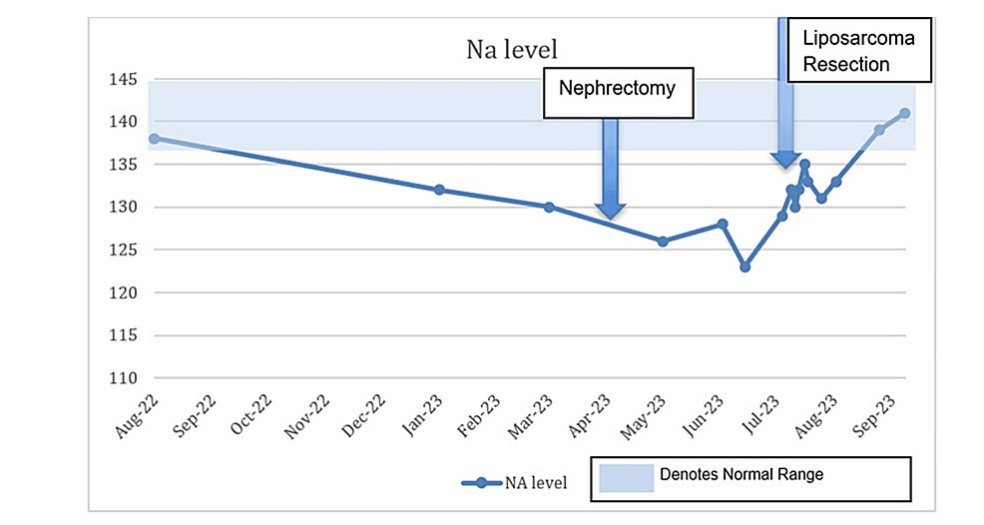
Sodium (Na) levels during hospitalization.

**Figure 4:**
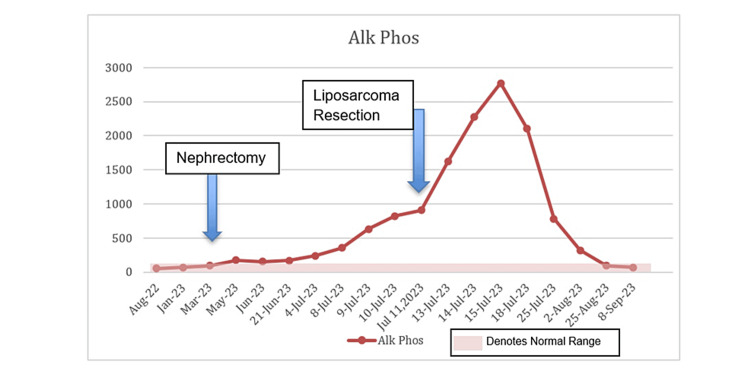
Alkaline phosphatase levels during hospitalization.

**Figure 5:**
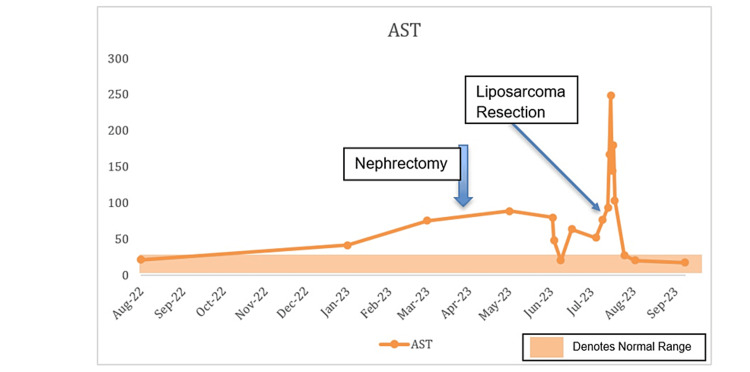
Trends in the levels of aspartate transaminase (AST) during hospitalization.

The source of the suspected sarcoma was hypothesized to have either been a de-differentiated spread from the clear cell RCC or a de novo sarcoma. Due to significant malnutrition and failure to thrive, which was felt related to an ongoing malignant process, the patient underwent a combined procedure with gastrostomy feeding tube placement and resection of the left thigh mass. Notable labs a day before the surgery were as follows: white blood cell count of 10.8 × 10^9^/L, hemoglobin of 7.8 g/dL, platelets of 344 × 10^9^/L, sodium of 129 mEq/L, ALP of 825 U/L, and AST of 65 U/L. The patient did not have jaundice during this time or scleral icterus but had gradually worsening anemia, persistent hyponatremia due to SIADH, and persistently elevated ALP and AST. The pathology from the thigh mass returned with findings of pleomorphic liposarcoma. The absence of *MDM2 *gene amplification suggested that this was not a de-differentiated tumor but more consistent with a de novo malignancy. The patient’s AST started to increase just before the RCC was discovered. After the patient’s nephrectomy, the ALP started to increase, and the ALP and the AST remained elevated until about two months after the nephrectomy when these values normalized. Thereafter, the ALP started to increase significantly, especially after the liposarcoma resection was performed. The AST remained elevated and spiked right after the liposarcoma resection. However, it was not until two months after the liposarcoma resection that both the ALP and the AST finally normalized, which was the first time that these were both normal since the original RCC was discovered. Regarding the hyponatremia, the sodium level remained low and only started to slowly increase and finally normalized about six weeks after the liposarcoma resection. Likewise, the patient’s serum albumin and hemoglobin levels significantly improved six to eight weeks after the liposarcoma resection. See Figure 3, Figure 4, and Figure 5 for trends of serum sodium, ALP, and AST, respectively.

The patient had coinciding liposarcoma with clear cell RCC that led to hepatic syndromes twice without liver metastasis and led to hyponatremia due to SIADH of malignancy. The other findings also supported that he had a variant of Stauffer syndrome without jaundice or icterus.

## Discussion

Stauffer syndrome is commonly a non-metastatic, nephrogenic, hepatic dysfunction syndrome that is linked to the effects invoked by paraneoplastic tumors [1,6]. Stauffer syndrome remains a rare occurrence that is yet to be completely unraveled. It can occur in RCC, but other malignancies have been associated with this syndrome [7]. Few studies have demonstrated the role of IL-6 in this syndrome. A study by Bhangoo et al. showed that cholestasis secondary to Stauffer syndrome could be related to the pro-inflammatory activity of IL-6 cytokines [8]. These effects were reversible when steroids were used in a prior case reported in the literature or when surgery removed the tumors, such as in our case [4]. These interventions seemed to be the most effective way of reversing the SIADH and hepatic syndrome. We hypothesize that during the liposarcoma tumor resection of our patient, there was a transient surge of IL-6 and other pro-inflammatory cytokines that led to a severe spike in ALP and AST after the procedure. This case emphasizes a unique and difficult clinical setting in which a patient simultaneously presented with clear cell RCC and liposarcoma, highlighting the need to take multiple diagnoses into account in patients with complicated medical histories. It also demonstrates the diagnostic difficulties in determining the etiology of liposarcoma in connection to the earlier RCC, demanding thorough diagnostic methods. Our patient with Stauffer syndrome also had SIADH which was felt to be caused by an IL-6 mechanism as well. The prevailing thought is that increased IL-6 produced by paraneoplastic tumors causes SIADH by binding both soluble and insoluble IL-6R which then activates transcription cascades that result in the release of vasopressin and affect the transporter gene production in hepatic biliary cells resulting in elevations in hepatic enzymes [2,3]. Additionally, the patient’s SIADH with hepatic syndrome, a variant of Stauffer syndrome, without liver metastases, highlights the need to detect paraneoplastic syndromes as critical diagnostic signals in cancer patients [9]. The fluctuating nature of lab results during the patient’s illness, such as hyponatremia and elevated platelet count, ALP, and AST levels, emphasizes the need for careful monitoring to guide therapy and evaluate treatment effectiveness. This case ultimately serves as a reminder of the significance of long-term patient care, follow-up, and possibly future therapy or interventions even after discharge, particularly in situations involving concurrent malignancies and related disorders [10]. From our case, we see that the patient’s labs worsened soon after the procedure but returned to normal levels a few days later. This could be attributed to either the surgical manipulation or the half-life of IL-6.

## Conclusions

Medical professionals should consider Stauffer syndrome in cases of unexplained cholestatic liver disease or unusual hepatic illnesses associated with paraneoplastic tumors. This syndrome may indicate a hidden malignant tumor not easily detected by standard methods, serving as an indirect diagnostic clue. Identifying Stauffer syndrome is paramount in the diagnostic process, especially when coupled with unexplained SIADH, as it may signal the presence of an undetected tumor or explain certain lab abnormalities. Therefore, maintaining a high level of clinical suspicion is crucial. In instances of liver function abnormalities deviating from typical patterns of liver disease, Stauffer syndrome should be included in the list of differential diagnoses.
